# Uncovering the active compounds and effective mechanisms of the dried mature sarcocarp of *Cornus officinalis* Sieb. Et Zucc. For the treatment of Alzheimer’s disease through a network pharmacology approach

**DOI:** 10.1186/s12906-020-02951-2

**Published:** 2020-05-25

**Authors:** Yan-Jie Qu, Rong-Rong Zhen, Li-Min Zhang, Chao Gu, Lei Chen, Xiao Peng, Bing Hu, Hong-Mei An

**Affiliations:** 1 Department of Neurology, Longhua Hospital, Shanghai University of Traditional Chinese Medicine, Shanghai, 200032 China; 2 Institute of Traditional Chinese Medicine in Oncology, Department of Oncology, Longhua Hospital, Shanghai University of Traditional Chinese Medicine, Shanghai, 200032 China; 3 Department of Science & Technology, Longhua Hospital, Shanghai University of Traditional Chinese Medicine, Shanghai, 200032 China

**Keywords:** Alzheimer’s disease, Chinese herb, *Cornus officinalis* Sieb. et Zucc., Network pharmacology

## Abstract

**Background:**

Shanzhuyu (the dried mature sarcocarp of *Cornus officinalis* Sieb. et Zucc., DMSCO) is a Chinese herb that can be used for the treatment of Alzheimer’s disease (AD), but its mechanism remains unknown. The present study aimed to investigate the active ingredients and effective mechanisms of DMSCO for the treatment of AD based on a network pharmacology approach.

**Methods:**

The active components of DMSCO were collected from the TCMSP and ETCM databases and the target proteins of these compounds were predicted using TCMSP, SwissTargetPrediction and the STITCH database. The AD-related target proteins were identified from the OMIM, DisGeNet, GEO and GeneCards databases. The network interaction model of the compound-target-disease was established and was used to obtain the key targets of DMSCO on AD through network topology analysis. Subsequently, gene enrichment in Gene Ontology (GO) and KEGG pathways were conducted using the David 6.8 online tool.

**Results:**

A total of 30 DMSCO effective compounds and 209 effective drug targets were obtained. A total of 172 AD-related genes and 37 shared targets of DMSCO and AD were identified. A total of 43 key targets for the treatment of AD were obtained from the topological analysis of the DMSCO-AD target network. These key targets were involved in a variety of biological processes, including amyloid deposition, apoptosis, autophagy, inflammatory response and oxidative stress and pathways, such as the PI3K-AKT, MAPK and TNF pathways. Three key compounds, namely ursolic acid, anethole and β-sitosterol were obtained from the analysis of the key targets.

**Conclusions:**

Ursolic acid, anethole and β-sitosterol may be the main active components of DMSCO in the treatment of AD. DMSCO can treat AD by regulating amyloid deposition, apoptosis, autophagy, inflammatory response and oxidative stress via the PI3K-AKT, MAPK and other signaling pathways.

## Background

Alzheimer’s disease (AD) is a neurodegenerative disease with clinical manifestations of progressive memory loss, cognitive impairment and personality changes. Currently, approximately 50 million people worldwide have dementia and more than 35% of the population over the age of 80 has this disease [1]. Approximately 5 million new cases of dementia are reported each year and the population is expected to grow to 118 million by 2050 [2]. The pathological feature of AD is loss of neurons, formation of senile plaques and neurofibrillary tangles involving the amyloid β (Aβ) and the tau protein, as well as oxidative stress and inflammation [3]. Studies that target Aβ and the tau protein are under investigation. However, they have not yielded satisfactory results [4, 5]. Current drugs, such as Donepezil and Memantine, are not effective to meet clinical needs. Therefore, it is necessary to study the treatment of AD from the perspective of multi-target therapy.

Traditional Chinese medicine (TCM) has been used to treat AD related diseases for thousands of years and significant clinical evidences have been accumulated. Shanzhuyu (the dried mature sarcocarp of *Cornus officinalis* Sieb. et Zucc., DMSCO) is a commonly used Chinese herb for the treatment of AD, which has the ability to tonify the liver and kidney. DMSCO is an important component of the Chinese herbal formula for AD-related disease treatment, such as Di-Huang-Yin-Zi, Liu-Wei-Di-Huang pills [6, 7]. DMSCO can inhibit Aβ_1–42_-induced apoptosis and inflammation, tau hyperphosphorylation and aggregation [8–10]. It can also inhibit cholinesterase and beta-site amyloid precursor protein cleaving enzyme 1 (BACE1) [11]. The active ingredients of DMSCO and their associated effective mechanism with regard to the treatment of AD require further investigation.

Network pharmacology is a new subject that has emerged recently. Network pharmacology can aid the exploration of the direct targets of the active ingredients of the Chinese herbs, define their functions in the context of molecular network [12]. In the present study, the active ingredients of DMSCO and its associated effective mechanism in the treatment of AD were systematically analyzed by establishing a “compound-target-pathway” network. The results indicated that DMSCO contained multiple active ingredients that could treat AD and that its mechanism was associated with the regulation of amyloid deposition, apoptosis, autophagy, inflammatory response and oxidative stress via the PI3K-AKT, MAPK and other signaling pathways.

## Methods

### Identification and screening of chemical ingredients of DMSCO

At present, several databases are available with regard to TCM ingredients. Traditional Chinese Medicine System Pharmacology (TCMSP, http://lsp.nwu.edu.cn/browse.php) is a comprehensive TCM platform containing 499 herbs and more than 2.9 × 10^4^ chemical components, providing comprehensive information on Chinese herbal ingredients, including chemical structure, oral bioavailability (OB), intestinal epithelial permeability, half-life, drug similarity and drug targets [13]. The Encyclopedia of Traditional Chinese Medicine (ETCM, http://www.nrc.ac.cn:9090/ETCM/) is also a commonly used TCM database, containing comprehensive information on Chinese herbs, TCM formulations and their ingredients [14]. TCMSP uses authoritative algorithms to predict the pharmacokinetic properties of compounds, such as absorption, distribution, metabolism and drug excretion (ADME) in order to provide comprehensive scores. In the present study, the chemical components of DMSCO were collected through literature research and via the TCMSP and ETCM databases. The ADME parameters OB ≥ 30% and Drug-likeness (DL) ≥ 0.18 were used to screen the potential active ingredients from the TCMSP database [15]. In addition, a Drug-likeness Weight ≥ 0.49 was used to retrieve the active ingredients of DMSCO from the ETCM database [14].

### Investigation and prediction of compound-related targets

Based on the chemical similarity and pharmacophore model, the present study used TCMSP, STITCH (http://stitch.embl.de/) and SwissTargetPrediction (http://www.swisstargetprediction.ch/) to retrieve and predict the related targets of compounds in DMSCO. STITCH is a database containing various structural and predictive interactions of compounds that support target prediction based on structural similarity [16]. In the present study, a confidence score ≥ 0.7 was used as the screening criterion. SwissTargetPrediction is a database used for predicting compound targets based on 2D and 3D structures of known compounds [17]. The probability value ≥ 0.5 served as the target screening standard in the present study.

### Identification of AD-related targets

AD-related genes were screened using online mendelian inheritance in man (OMIM, https://omim.org/) [18], DisGeNET (http://www.disgenet.org/) [19], GeneCards (https://www.genecards.org/) [20], and Gene Expression Omnibus (GEO) databases (http://www.ncbi.nlm.nih.gov/geo). DisGeNET is a comprehensive platform developed to solve problems regarding the genetic basis of human disease. The platform was searched using the keyword “Alzheimer’s Disease” and disease related genes were identified based on a score ≥ 0.4. AD-related genes with GeneCards Inferred Functionality Score (GIFtS) ≥ 52 were selected from the GeneCards database. We further used the GEO2R online tool (http://www.ncbi.nlm.nih.gov/geo/geo2r/) to select AD-related genes from the GSE36980 dataset (15 AD patients and 33 healthy subjects). The criteria for screening differentially expressed genes were *P* ≤ 0.05, fold change (FC) ≥ 1.5. The target ID was converted to the gene symbol by retrieving either the UniProtKB (https://www.uniprot.org/) or the STRING (https://string-db.org/) databases.

### Network construction and topological analysis

PPI (protein-protein network) was constructed via the STRING database and the targets with a confidence score ≥ 0.7 were selected. The following network and topology analyses were performed using the Cytoscape 3.6.0 software: 1. The compound-target network of DMSCO; 2. The AD-related target network; 3. The DMSCO potential target-AD target interaction network; 4. The networks of shared targets between DMSCO and AD targets. Degree centrality (DC), betweenness centrality (BC) and closeness centrality (CC) are the most common topology parameters used to evaluate the central properties of nodes in a network. In the DMSCO potential target-AD target interaction network, the parameter settings of DC ≥ 3 × median DC, BC ≥ median BC and CC ≥ median CC were used to screen the key targets of DMSCO.

### GO and KEGG pathway enrichment analysis

DAVID 6. 8 (https://david.ncifcrf.gov/) is an online biological information repository and analysis tool for extracting biological information regarding gene functional annotation and pathways enrichment [21]. Drug targets and key targets of DMSCO acting on AD were imported into the DAVID 6.8 database and the species were defined as “*Homo sapiens*”, whereas the target genes were identified as “official gene symbol”. Gene Ontology (GO) and KEGG pathway analysis were performed.

## Results

### DMSCO compound-target network

According to the search results of TCMSP, DMSCO exhibited a total of 226 chemical components, including mainly iridoids, pentacyclic triterpenoid acids and their corresponding esters, polysaccharides and tannins. A total of 20 compounds were screened by OB ≥ 30% and DL ≥ 0.18. A total of 55 types of compounds were retrieved from ETCM and 20 compounds were filtered by Drug-likeness Weight ≥ 0.49. A thorough literature search using Chinese and international references resulted in the identification of 7 important active ingredients that fulfilled the aforementioned standards. Therefore, 47 compounds were obtained in total. TCMSP, STITCH and SwissTargetPrediction were used to collect and predict targets and 17 compounds were identified without a corresponding target. Therefore, 30 DMSCO effective compounds were obtained, corresponding to 209 targets.

**Fig. 1:**
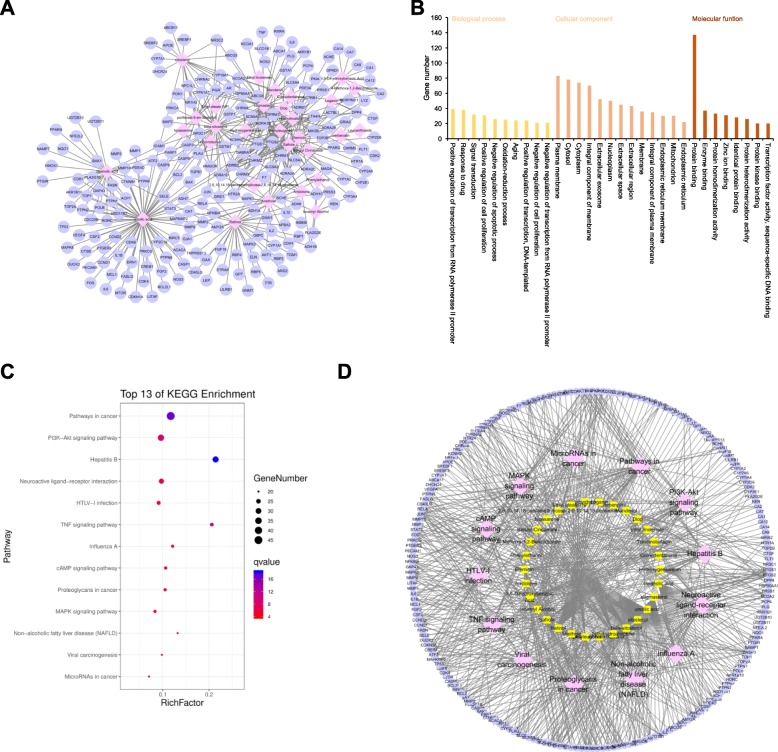
Potential targets of DMSCO compounds. **a** DMSCO Compound-target network was generated by Cytoscape 3.6.0 software. It consists of 239 nodes and 435 edges. Pink diamond nodes represent compounds in DMSCO, while lavender round nodes represent potential targets of DMSCO. **b** GO enrichment analysis for potential targets of DMSCO (Count number > 15). **c** KEGG pathway enrichment analysis for potential targets of DMSCO (Count number ≥ 12), q value refers to -log10(*P* value). **d** DMSCO compound-target-pathway (Count number > 20) network were generated by Cytoscape 3.6.0 software. Lavender eclipse nodes stand for potential targets of DMSCO, while yellow round rectangle nodes stand for active compounds in DMSCO and pink diamond nodes stand for the pathways whose count number > 20

The main information of the compounds with the number of targets > 10, namely the main active components of DMSCO are listed in Table 1. Among these major active ingredients, ursolic acid exhibited a total of 72 targets, whereas β-sitosterol exhibited 39 targets, stigmasterol 38 targets, retinol 33 targets and tetrahydroalstonine 30 targets. Safrole, methyleugenol, oleanolic acid, elemicin, anethole, sitosterol and poriferast-5-en-3beta-ol (clionasterol) demonstrated 27, 26, 25, 22, 21, 19 and 12 targets, respectively. The corresponding association between compounds and targets is shown in Fig. 1a.

**Table 1:** The characteristics of active compounds in DMSCO

Compounds	Molecular formula	Molecular weight	OB (%)	DL
Ursolic acid	C_30_H_48_O_3_	456.78	16.77	0.75
Beta-sitosterol	C_29_H_50_O	414.79	36.91	0.75
Stigmasterol	C_29_H_48_O	412.77	43.83	0.76
Retinol	C_20_H_30_O	286.5	19.53	0.16
Tetrahydroalstonine	C_21_H_24_N_2_O_3_	352.47	32.42	0.81
Safrole	C_10_H_10_O_2_	162.2	45.34	0.05
Methyleugenol	C_11_H_14_O_2_	178.25	73.36	0.04
Oleanolic acid	C_30_H_48_O_3_	456.78	29.02	0.76
Elemicin	C_12_H_16_O_3_	208.28	21.94	0.06
Anethole	C_10_H_12_O	148.22	32.49	0.03
Sitosterol	C_29_H_50_O	414.79	36.91	0.75
Poriferast-5-en-3beta-ol	C_29_H_50_O	414.79	36.91	0.75

*OB* Oral bioavailability, *DL* Drug-likeness

In order to clarify the characteristics of the relevant targets of the key active components of DMSCO, GO and KEGG pathway enrichment analyses were performed (Fig. 1b and c). These targets existed in the nucleus, cytoplasm and plasma membrane of cell and were involved in biological processes, such as transcriptional regulation, drug response, signal transduction, cell proliferation and senescence. The molecular function of these genes was involved in binding proteins, enzymes and zinc ions. KEGG enrichment analysis indicated that 127 pathways were affected by the active components of DMSCO (*P* < 0.05). The top 13 (Count Number ≥ 12) pathways included cancer, hepatitis B, PI3K-AKT, neuroactive ligand-receptor interactions, HTLV-I infection, TNF, influenza A, cAMP, proteoglycans in cancer, MAPK, non-alcoholic fatty liver disease, viral carcinogenesis and microRNAs in cancer signaling pathways. Based on this information, the network association of DMSCO compound-target-pathway was established (Fig. 1d).

### AD-target network

**Fig. 2:**
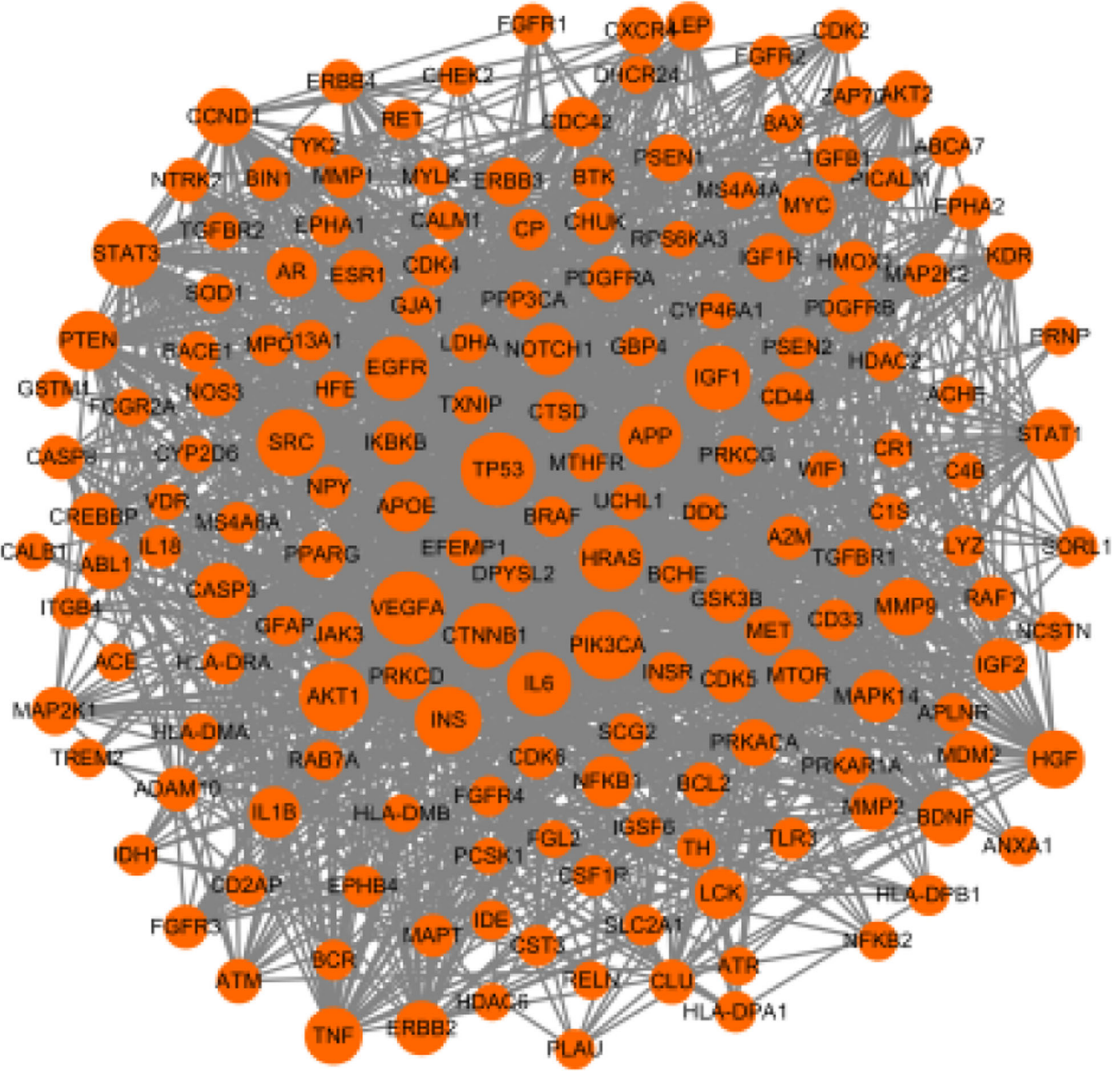
AD-target network. AD-target network was generated by Cytoscape 3.6.0 software. Orange round nodes represent AD-related targets, and the size of nodes is proportional to degree centrality by topology analysis

A total of 218 AD related genes were screened from OMIM, DisGeNET, GeneCards and GEO databases, among which 172 targets exhibited high interaction in the protein-protein interaction (PPI) graph generated from the STRING database (confidence score ≥ 0.7). The AD disease target network was constructed using Cytoscape 3.6.0, consisting of 172 nodes and 1410 edges and the central properties of each node were evaluated using topology analysis (Fig. 2). The size of nodes was proportional to the degree centrality obtained from topology analysis. The results indicated that TP53, VEGFA, PIK3CA, AKT1, SRC, STAT3, INS, IGF1, HRAS, CTNNB1, IL6, EGFR, APP and PTEN were important target genes of AD (degree > 40).

### DMSCO target-AD target network

**Fig. 3:**
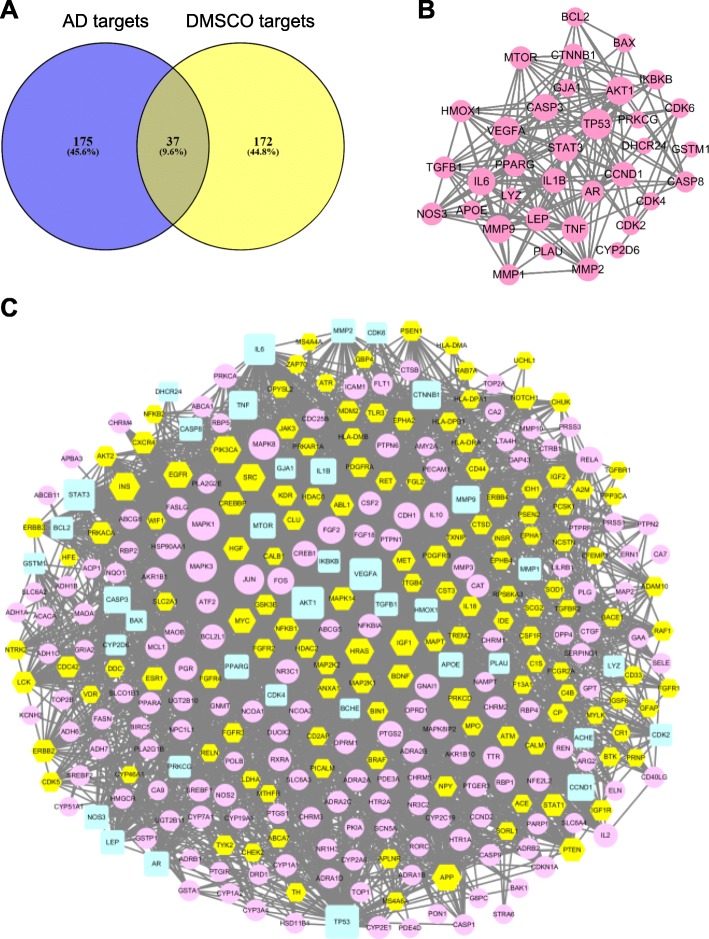
Shared targets between AD targets and DMSCO targets. **a** Shared targets between AD targets and DMSCO targets were identified by Venny 2.1. **b** Network of the shared targets was generated by Cytoscape 3.6.0 software. Pink round nodes represent the shared targets, and the size of nodes is proportional to degree centrality by topology analysis. **c** DMSCO target-AD targets network was generated by Cytoscape 3.6.0 software. Yellow hexagon nodes stand for DMSCO targets, pink eclipse nodes represent AD-related targets, and light blue round rectangle nodes represent the shared targets of DMSCO and AD. The size of nodes is proportional to degree centrality by topology analysis

In order to determine the association between DMSCO potential targets and AD disease related targets, the Venn online tool (https://bioinfogp.cnb.csic.es/tools/venny/index.html) was used to obtain 37 shared targets of DMSCO and AD (Fig. 3a). The interaction network between these genes was established and 35 targets indicated high interaction (confidence score ≥ 0.7) in the PPI diagram generated from the STRING database (Fig. 3b). The network consists of 35 nodes and 189 edges and the size of nodes is proportional to degree centrality obtained from topology analysis. Topology analysis indicated that TP53, VEGFA, AKT1, MMP9, IL6, STAT3, TNF, CASP3, CCND1 and IL1B were the top 10 shared targets from the perspective of degree centrality. Subsequently, we established the interaction network of DMSCO target-AD targets (Fig. 3c), which was composed of 331 nodes and 3226 edges. The node size was proportional to degree centrality obtained from topology analysis.

### Key targets of the DMSCO-AD interaction network

**Fig. 4:**
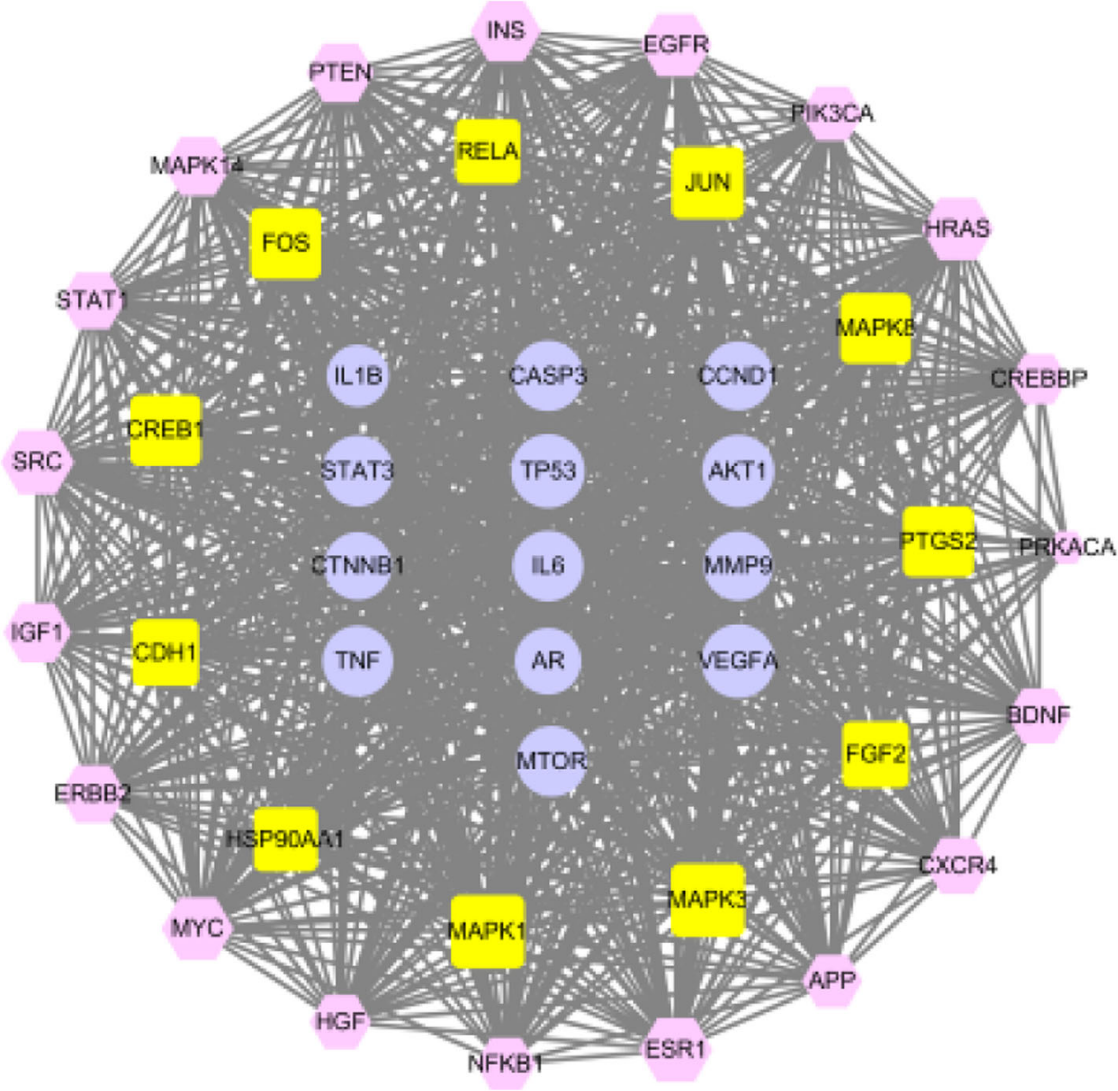
Network of key targets. Key targets were identified by central network evaluation and its network was generated by Cytoscape 3.6.0 software. Pink hexagon nodes stand for DMSCO targets, yellow round rectangle nodes represent AD-related targets, and lavender eclipse nodes represent the shared targets of DMSCO and AD. The size of nodes is proportional to degree centrality by topology analysis

**Fig. 5:**
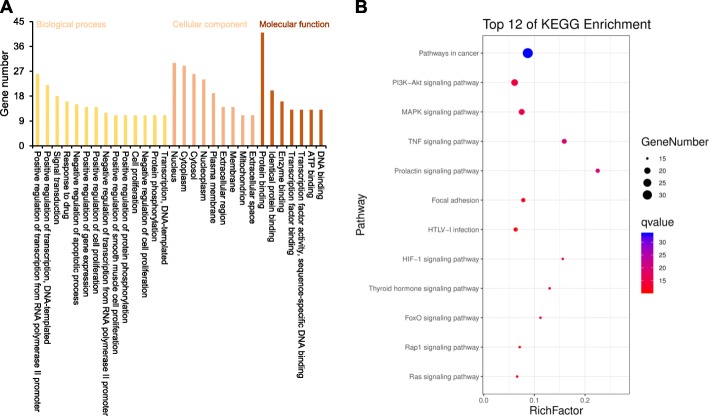
GO and KEGG enrichment analysis of key targets. **a** GO enrichment analysis of key targets (count number > 10). **b** KEGG pathway enrichment analysis of key targets (count number ≥ 15), q value refers to -log10(*P* value)

**Fig. 6:**
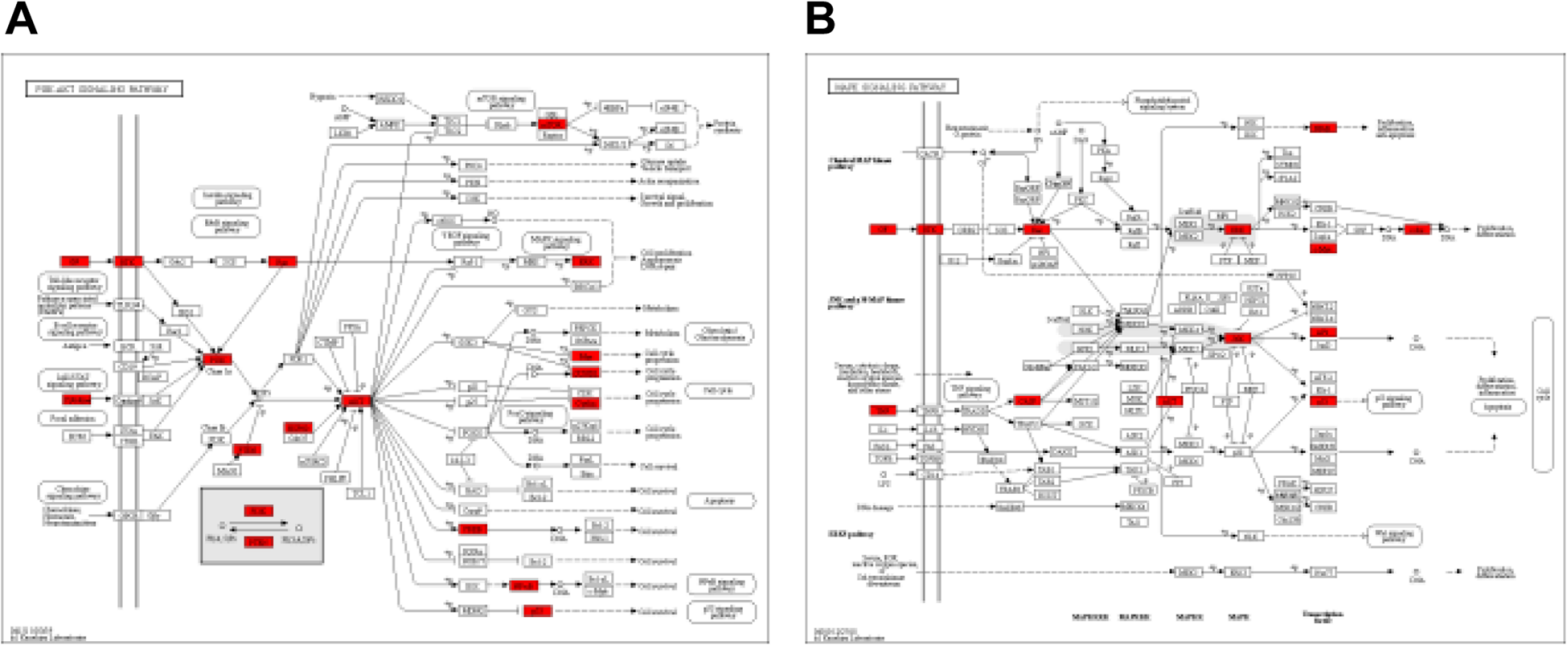
Distribution of key targets in the most enriched pathways. **a** Distribution of key targets in the PI3K/AKT signaling pathway. **b** Distribution of key targets in the MAPK signaling pathway. The red rectangle stands for the key targets

In the DMSCO target-AD target interaction network, 43 key targets and 814 interactions were obtained through central network evaluation (Fig. 4). The information of key targets of degree centrality ≥ 50 was listed in Table 2. GO enrichment analysis indicated that these targets mainly existed in the nucleus, cytoplasm, plasma membrane and other regions of cell and were involved in transcriptional regulation, apoptosis, signal transduction, drug response, cell proliferation, gene expression, protein phosphorylation and other biological processes. They exhibited various molecular functions, such as binding proteins, enzymes, ATP, DNA and transcription factors (Fig. 5a). KEGG pathway enrichment analysis indicated that 119 pathways were affected by the active component of DMSCO (*P* < 0.05). The top pathways (count number ≥ 15) included cancer, PI3K-AKT, MAPK, TNF, focal adhesion, prolactin, HIF-1, thyroid hormone, FoxO, Rap1 and Ras signaling pathways (Fig. 5b). The distribution of key targets in the PI3K-AKT and MAPK pathways is shown in Fig. 6.

**Table 2:** Top 30 key targets of DMSCO acting on AD (degree ≥ 50)

Targets	Official name	Degree	BC	CC
TP53	Tumor protein p53	102	0.082	0.557
MAPK1	Mitogen-activated protein kinase 1	95	0.037	0.539
AKT1	AKT serine/threonine kinase 1	93	0.029	0.533
VEGFA	Vascular endothelial growth factor A	90	0.033	0.538
INS	Insulin	86	0.087	0.547
IL6	Interleukin 6	86	0.047	0.54
STAT3	Signal transducer and activator of transcription 3	85	0.017	0.519
PIK3CA	Phosphatidylinositol-4,5-Bisphosphate 3-Kinase Catalytic Subunit Alpha	84	0.037	0.519
MAPK8	Mitogen-activated protein kinase 8	83	0.029	0.529
MAPK3	Mitogen-activated protein kinase 3	83	0.027	0.532
SRC	SRC Proto-Oncogene, Non-Receptor Tyrosine Kinase	81	0.027	0.513
APP	Amyloid beta precursor protein	77	0.072	0.514
JUN	Jun proto-oncogene, AP-1 transcription factor subunit	77	0.028	0.532
EGFR	Epidermal growth factor receptor	74	0.027	0.518
HRAS	HRas Proto-Oncogene, GTPase	70	0.014	0.503
IGF1	Insulin like growth factor 1	70	0.013	0.511
CTNNB1	Catenin beta 1	66	0.033	0.498
TNF	Tumor necrosis factor	66	0.011	0.505
MYC	MYC proto-oncogene, bHLH transcription factor	62	0.012	0.498
MMP9	Matrix metallopeptidase 9	59	0.024	0.498
FGF2	Fibroblast growth factor 2	58	0.01	0.495
FOS	Fos proto-oncogene, AP-1 transcription factor subunit	54	0.015	0.498
CASP3	Caspase 3	54	0.011	0.492
CCND1	Cyclin D1	54	0.004	0.475
PTEN	Phosphatase and tensin homolog	53	0.005	0.474
RELA	RELA proto-oncogene, NF-kB subunit	52	0.007	0.487
HGF	Hepatocyte growth factor	51	0.006	0.484
BDNF	Brain derived neurotrophic factor	50	0.021	0.498
CDH1	Cadherin 1	50	0.013	0.487

*BC* Betweenness centrality, *CC* Closeness centrality

The key targets related to each compound were listed in Table 3. Ursolic acid, anethole and β-sitosterol were related to the largest number of key targets, including 17, 7 and 5 key targets, respectively. These results suggested that ursolic acid, anethole and β-sitosterol were the main compounds in DMSCO that were most closely associated with the treatment of AD.

**Table 3:** DMSCO compounds and related key targets

Compounds	Number of key targets	Targets
Ursolic acid	17	MAPK8, JUN, FGF2, FOS, RELA, PTGS2, CREB1, TP53, VEGFA, IL6, STAT3, CTNNB1, MMP9, CASP3, CCND1, IL1B, MTOR
Anethole	7	MAPK1, MAPK3, JUN, RELA, CDH1, AKT1, MMP9
Beta-sitosterol	5	JUN, HSP90AA1, PTGS2, CASP3, AR
Stigmasterol	3	PTGS2, TNF, AR
Tetrahydroalstonine	3	HSP90AA1, PTGS2, AR
Retinol	2	MAPK1, JUN
Sitosterol	2	CASP3, AR
Poriferast-5-en-3beta-ol	2	CASP3, AR
Hydroxygenkwanin	2	HSP90AA1, PTGS2
Safrole	2	PTGS2, CASP3
Oleanolic acid	1	CASP3
Methyleugenol	1	PTGS2
Mandenol	1	PTGS2
3,5-Dihydroxybenzoic Acid	1	PTGS2
Aristolone	1	PTGS2
Verbenalin	1	PTGS2
Elemicin	1	PTGS2
Cornudentanone	1	PTGS2
2,6,10,14,18-pentamethylicosa-2,6,10,14,18-pentaene	1	PTGS2

It is worth noting that the results of enrichment and pathway analysis of drug targets of DMSCO and key targets of the DMSCO-AD interaction network are not completely consistent. By comparison, it was found that the major biological processes of DMSCO drug targets are mRNA transcription regulation, drug response and signal transduction, while the major biological processes of key targets are mRNA transcription regulation, DNA-templated and signal transduction. The cellular components of DMSCO drug targets are mainly plasma membrane, cytosol and cytocomponents, while the cellular components of key targets are mainly nucleus, cytoplasm and cytosol. The molecular functions of DMSCO drug targets are mainly protein binding, enzyme binding and protein homodimerization activity, while the molecular functions of key targets are protein binding, enzyme binding and protein-like binding. These results suggested that drug targets may have a wider range of action than key targets. Key targets are the parts of the DMSCO drug targets that specifically treats AD. In other words, key targets have a more specific treating direction. As for the pathway, key targets mainly focused on PI3K-Akt and MAPK signaling pathway, while the drug targets are mainly enriched in hepatitis B and neuroactive ligand-receptor interaction in addition to these two pathways. These results suggested that in addition to AD, DMSCO may has effects on other diseases, such as hepatitis B, which is worth further study.

## Discussion

In the present study, network pharmacology was used to explore the key compounds and potential targets of DMSCO for the treatment of AD, providing a basis for the development of a feasible alternative therapy for AD. The results indicated that ursolic acid, polysitosterol, cornuside, sweroside, morroniside, loganin, garlic acid, stigmasterol, retinol, tetrahydroalstonine, oleanolic acid, anethole, methyleugenol and other compounds were the possible effective components of DMSCO responsible for its anti-AD effect. Ursolic acid, anethole and β-sitosterol were the key compounds in DMSCO that could be used for the treatment of AD.

Ursolic acid inhibits the extracellular signaling regulation kinases, p38 and JNK, leading to the inactivation of NF-κB and to the downregulation of COX-2 and iNOS, which are induced by Aβ_1–42_ peptides. This in turn leads to the production of anti-inflammatory and neuroprotective effects [22]. β-sitosterol exhibits potent anticholinesterase and antioxidant activities, which can improve memory and learning impairment in APP/PS1 double-transgenic AD mice [23, 24]. Anethole can reduce hypoxia-glucose deprivation-reoxygenation-induced nerve cell death and its mechanism is associated with antioxidant activity, antiexcitatory toxicity and mitochondrial protection [25]. Chainy et al. demonstrated that anethole could block the early and late cellular responses mediated by TNF-α, while also affecting the NF-κB, AP-1, JNK and MAPK pathways and inhibiting the H_2_O_2_-induced activation of NF-κB [26].

In addition to these three key compounds, other components also possess anti-AD effects. Loganin exhibits anti-inflammatory and memory improvement effects and can inhibit Aβ_1–42_-induced microglial activation and inflammatory response by inhibiting TLR4/TRAF6/NF-κB signal transduction [10]. Morroniside plays a neuroprotective role by increasing the activity of PP2A and inhibiting phosphorylation of tau protein at multiple sites including Thr217 [27]. Oleanolic acid inhibits the secretion of Aβ-activated inflammatory cytokines, such as IL-6, TNF-β and IL-1β and alleviates the neuronal apoptosis caused by Aβ, thus improving the cognitive deficits in rats with AD [28].

In the present study, 43 key targets of DMSCO were identified for AD treatment and the top 10 targets were TP53, MAPK1, AKT1, VEGFA, INS, IL6, STAT3, PIK3CA, MAPK8 and MAPK3. TP53, also known as p53, is a transcription factor that can initiate apoptosis. The expression and activity of p53 increase rapidly following hypoxia, DNA damage, oncogene activation, microtubule destruction and oxidative damage conditions [29], while p53-mediated apoptosis is associated with chronic neuronal degeneration. Neuregulin-1 can significantly reduce CoCl_2_-induced SH-SY5Y cell death by regulating HIF-1α and p53 [30]. It has been proposed that the detection of the Aβ42 peptide, tau, p16 and p53 protein expression levels is a promising method for diagnosing AD and evaluating the aging rate during the development of this disease [31]. VEGFR-2 plays a key role in angiogenesis and is involved in the development of the central nervous system. Aβ1–42 participates in the pathogenesis of AD by inhibiting VEGF-induced endothelial cell migration and VEGFR-2 activity [32]. AD is also associated with the JAK-STAT pathway [33]. Curcumin can regulate the inflammatory response of microglial cells through JAK/STAT/SOCS signaling, thus regulating the neuroinflammatory response [34].

The pathological of AD is closely associated with oxidative stress, the inflammatory response and Aβ deposition. The present study demonstrated that DMSCO regulated AD-related pathways, such as the PI3K/AKT and MAPK pathways. The PI3K-AKT pathway is an important pathway that mediates neuronal survival. It can lead to autophagy disruption and slow down AD progression [35]. The PI3K/AKT/mTOR signals inhibit apoptosis and autophagy, regulate oxidative stress and play a neuroprotective role in SH-SY5Y cells treated with Aβ_1–42_ [36]. Deuterium-depleted water can inhibit H_2_O_2_-induced oxidative stress by upregulating the PI3K/AKT signaling [37]. The MAPK pathway mediates the inflammatory response induced by Aβ_1–42_ [38]. EGb761 suppresses the neuroinflammatory response induced by Aβ_1–42_ in virtue of inhibiting the phosphorylation of MAPK [39]. It has also been shown that miR330 inhibits oxidative stress damage in AD mice and alleviates mitochondrial dysfunction by regulating MAPK signal transduction, which reduces the production of Aβ [40].

## Conclusions

In summary, the present study demonstrated that ursolic acid, anethole and β-sitosterol may be the main active ingredients of DMSCO in the treatment of AD. DMSCO can treat AD by regulating amyloid deposition, apoptosis, autophagy, inflammatory response and oxidative stress via the PI3K-AKT, MAPK and other signaling pathways. The present study provides a basis for the treatment of AD by DMSCO from the perspective of network pharmacology. We identified the main components, targets and pathways of DMSCO in the treatment of anti-AD, these findings have guiding significance for application of DMSCO, and provide new clues for the future study and development of anti-AD drugs.

## Data Availability

The datasets used and/or analyzed during the current study available from the corresponding author on reasonable request.

## Abbreviations

AD: Alzheimer’s disease
Aβ: Amyloid β
ADME: Absorption, distribution, metabolism and drug excretion
BACE1: Beta-site amyloid precursor protein cleaving enzyme 1
BC: Betweenness centrality
CC: Closeness centrality
COX-2: Cyclooxygenase-2
DC: Degree centrality
DL: Drug-likeness
DMSCO: The dried mature sarcocarp of *Cornus officinalis* Sieb. et Zucc.
ETCM: Encyclopedia of traditional Chinese medicine
FC: Fold change
GEO: Gene expression omnibus
GO: Gene ontology
iNOS: Inducible nitric oxide synthase
JNK: c-Jun N-terminal kinase
KEGG: Kyoto encyclopedia of genes and genomes
MAPK: Mitogen-activated protein kinase
NF-κB: Nuclear factor kappa B
OB: Oral bioavailability
OMIM: Online Mendelian inheritance in man
PI3K: Phosphoinositide 3-kinase
PPI: Protein-protein network
TCMSP: Traditional Chinese medicine system pharmacology
TNF-α: Tumor Necrosis Factor α
VEGFR: Vascular endothelial growth factor receptor
